# Enhancing Catalytic Removal of *N*-Nitrosodimethylamine from Drinking Water Matrices with One-Step-Carbonized Ferric Ammonium Citrate

**DOI:** 10.3390/nano15110831

**Published:** 2025-05-30

**Authors:** Jing Lv, Lingyue Zhang, Jialu Li, Yuting Zhang, Ruofan Wang, Rui Tang, Jianchao Wang, Mei Hong, Na Liu

**Affiliations:** 1 Key Laboratory of Groundwater Resources and Environment, College of New Energy and Environment, Jilin University, Ministry of Education, Changchun 130021, China; lvjing19@mails.jlu.edu.cn (J.L.); jialu18@mails.jlu.edu.cn (J.L.); wangrf96@gmail.com (R.W.); tangrui21@mails.jlu.edu.cn (R.T.); hongmei@jlu.edu.cn (M.H.); 2 Department of Civil Engineering, The University of Hong Kong, Hong Kong 999077, China; 3 Key Laboratory of Songliao Aquatic Environment, Jilin Jianzhu University, Ministry of Education, Changchun 130118, China; zhangyuting@jlju.edu.cn; 4 State Key Laboratory of Nutrient Use and Management, College of Resources and Environmental Sciences, National Academy of Agriculture Green Development, Key Laboratory of Plant–Soil Interactions, China Agricultural University, Ministry of Education, Beijing 100193, China; jcwang91@cau.edu.cn; 5 Department of Ecology, College of Life Science and Technology, Jinan University, Guangzhou 510632, China

**Keywords:** advanced oxidation processes (AOPs), *N*-nitrosodimethylamine (NDMA), pyrolytic carbonization, ammonium ferric citrate, superoxide radical

## Abstract

N-Nitrosodimethylamine (NDMA) is a widely recognized disinfection by-product that poses significant carcinogenic risks in drinking water. Conventional methods for NDMA removal, such as nanofiltration and reverse osmosis membranes, have limited efficacy due to NDMA’s small molecular weight and polar properties. Advanced oxidation processes (AOPs) have shown promise, but traditional Fenton processes often fall short due to the chemical structure of nitrosamines in NDMA. This study proposes a novel, cost-effective approach using a one-step carbonization method to synthesize a catalyst from ferric ammonium citrate (FAC). The resulting FAC-600 integrates zero-valent iron and iron carbide with carbon-based functional groups, enhancing catalytic and electron transport activities. Our experiments demonstrated that the FAC-600/persulfate (PS) AOP system achieves over 90% NDMA removal across a wide concentration range (50 μg L^−1^ to 1000 μg L ^−1^) with a limited dosage of 0.5 g L^−1^. Mechanistic insights revealed that superoxide and hydroxyl radicals dominate NDMA degradation, facilitated by the presence of dissolved oxygen and PS. This study underscores the potential of the FAC-600/PS AOP system as a robust and efficient solution for NDMA removal, promising safer drinking water through practical application.

## 1. Introduction

N-Nitrosodimethylamine (NDMA) is a widely recognized disinfection by-product that is highly soluble in water [1]. NDMA is the most commonly detected nitrosamine and has been well documented as a carcinogen [2]. It has a reported [1] lifetime cancer risk of 10^−6^ with a drinking water equivalent concentration of 0.7 ng L^−1^. Due to its small molecular weight (74.08 g mol^−1^) and polar properties [3], the removal of NDMA from practical drinking water matrices by conventional nanofiltration (NF) and reverse osmosis (RO) membranes remains limited [4]. There is great demand for the removal of NDMA through robust and cost-efficient processes [5].

Conventional reduction reactions can only reduce NDMA to dimethylamine (DMA) or unsymmetrical dimethylhydrazine (UDMH, Figure S1), making complete removal challenging [6]. Recently, advanced oxidation processes (AOPs) have been considered a feasible approach for the efficient treatment of NDMA [7]. The use of ozone/hydrogen peroxide (O_3_/H_2_O_2_) is a commonly applied AOP for the oxidative degradation of NDMA, requiring 160–320 μM ozone to achieve 50–75% NDMA oxidation [3]. Ultraviolet/hydrogen peroxide (UV/H_2_O_2_) has also been widely implemented for NDMA removal, with high rate constants of reactions between NDMA and hydroxyl radicals [8]. However, due to the relatively stable functional group structure of nitrosamines, traditional Fenton processes have a limited ability to oxidize and degrade NDMA [9].

Persulfate (PS)-based AOPs offer several advantages, including higher oxidation potential, greater selectivity and efficiency in oxidizing pollutants, and applicability over a wider pH range [10]. Various materials, such as activated carbon, transition metals, and graphene oxide, have been reported for the activation of PS to form radicals capable of effectively degrading emerging pollutants [11]. Iron and its oxides are among the most studied metals due to their environmental friendliness, relative non-toxicity, and cost-effectiveness compared to other transition metals [12,13]. Zero-valent iron (ZVI) is one of the most effective catalysts, demonstrating high pollutant removal capacity with its redox potential [14]. However, ZVI’s strong reducibility leads to rapid oxidation during the degradation process, and the interaction between a large number of free radicals can affect pollutant removal efficiency [15]. Additionally, the fabrication of ZVI is complex and costly [16].

To develop a feasible strategy for removing NDMA from practical drinking water matrices, this study designs a one-step carbonization method for ferric ammonium citrate (FAC) to enhance the catalytic removal of NDMA. Through this feasible process, ZVI and iron carbide (Fe_3_C) are fabricated with carbon-based functional groups. This integrated structure enables efficient catalytic and electron transport activity. Material characterization and free radical quenching experiments were conducted to elucidate the dominant degradation mechanism.

## 2. Materials and Methods

### 2.1. Materials

Ferric ammonium citrate (C_12_H_22_FeN_3_O_14_, Fe ≥ 16.5–18.5%), disodium hydrogen phosphate (Na_2_HPO_4_, >99.5%), sodium dihydrogen phosphate (NaH_2_PO_4_, >99.5%), sodium phosphate (Na_3_PO_4_, >99%), and sodium sulfate (Na_2_SO_4_, 99.5%) were purchased from Damao (Tianjin, China). Hydrochloric acid (HCl, 36–38%), sodium hydroxide (NaOH, >98%), and NDMA (C_2_H_6_N_2_O) were purchased from Sigma-Aldrich (Shanghai, China). Sodium persulfate (Na_2_S_2_O_8_, 99%), methanol (HPLC grade, 99.9%), sodium chloride (NaCl, ≥99.5%), and sodium bicarbonate (NaHCO_3_, ≥99.5%) were purchased from Macklin (Shanghai, China). DMA (C_2_H_7_N, standard solution) and UDMH (C_2_H_8_N_2_, standard solution) were purchased from Weiyejiliang (Beijing, China). Humic acid (HA, ≥97%) was purchased from Bosf (Hefei, China). ZVI (≥99.5%) was purchased from North China Science and Technology Metal Materials (Hebei, China). Fe_3_C (≥99.5%) was purchased from Fenghui Nanometer Technology Enterprise Store (Hebei, China). The ultrapure water used to prepare the solution was prepared by the GWB Series ultrapure water machine.

### 2.2. Synthesis of Carbonized FAC Nanomaterials

To prepare carbonized FAC nanomaterials, a one-step method was employed. Before fabrication, 2.0 g of FAC was dried in a blast furnace at 60 °C overnight. A carbonization temperature of 600 °C was selected based on preliminary experimental results (Figure S2). The dried FAC powder was then subjected to pyrolytic carbonization at 600 °C with a heating rate of 8 °C min^−1^ in a tube furnace under a nitrogen atmosphere for 120 min. The resulting FAC-600 powder was stored directly in an anaerobic glove box.

### 2.3. Batch Experiment

All reactions were carried out in a 60 mL headspace vial. To start the experiment, 20 mg of FAC-600 was added to 32 mL of NDMA solution (500 μg L^−1^), followed by 8 mL of PS solution (0.8 mmol L^−1^). The reaction was oscillated at 25 °C in a water bath oscillator, at a speed of 150 rpm.

To explore the removal capacity of FAC-600, experiments using various NDMA concentrations from 50 μg L^−1^ to 20 mg L^−1^ were also conducted. Samples were taken at specific times (0, 20, 40, 60, 90, 120, 150, 180, 210, 240 min) for the analysis of reduction kinetics. The removal rate of NDMA by sodium persulfate was investigated at a concentration of 0.1–1.2 mmol L^−1^. The pH of the solution was adjusted using 0.1 M NaOH and 0.1 M HCl for the initial pH value effect experiment and phosphate buffer pairs for the buffer pH value effect experiment, and monitored using a pH meter (T50, Mettler Toledo, Switzerland). All the experiments were carried out three times in parallel (the pre-experiment determined that the experimental container and the sampling container have no adsorption effect on NDMA and its degradation products).

Kinetics data were fitted with the pseudo-first-order model, as shown in the following equation:

ln(C_t_/C_0_) = −k_1_t,

where C_t_ (mg L^−1^) is the concentration of NDMA at t min, C_0_ (mg L^−1^) is the initial concentration of NDMA, k_1_ (min⁻^1^) is the rate constant of the pseudo-first-order model, and t (min) is the reaction time.

### 2.4. Analytical Methods

For the determination of NDMA, the experimental liquid samples were filtered through a 0.22 μm water filter membrane and placed in Agilent vials. The samples were then analyzed using an Agilent 1260 Infinity II liquid chromatograph (UPLC; Agilent, Santa Clara, CA, USA) equipped with a UV detector. The detection wavelength was set at 230 nm. A C8 column (2.7 μm, 4.6 × 100 mm) was utilized, with a mobile phase composed of methanol and water in a 5:95 ratio. The flow rate was maintained at 0.6 mL/min, and the injection volume was 100 μL.

For the determination of UDMH, the experimental liquid samples were derivatized with 4-nitrobenzaldehyde. Post-derivatization, the samples were analyzed using the same liquid chromatograph. The UV detector was set to a wavelength of 390 nm, and a C8 chromatographic column was used. The mobile phase consisted of methanol and water in an 80:20 ratio, with a flow rate of 0.6 mL/min. The injection volume was 100 μL.

For the determination of DMA, the experimental liquid samples were derivatized with benzenesulfonyl chloride. Following derivatization, the samples were analyzed using gas chromatography–mass spectrometry (GC-MS) with an Agilent 6890 GC 5973 MS system. The GC method parameters were as follows: The chromatographic column was DB-5MS (length: 30 m; inner diameter: 0.25 mm; film thickness: 0.25 μm). The inlet temperature was set at 280 °C, with a split injection mode at a split ratio of 3:1 and a split flow rate of 3 mL min^−1^. The column temperature program was as follows: 120 °C for 3 min, ramped to 220 °C at 5 °C min^−1^, and then increased to 290 °C at 10 °C min^−1^, holding for 5 min. The injection volume was 1 μL. The MS method operated in selected ion mode (SIM), with selected ions at m z^−1^ 77.10 and 185.10.

### 2.5. Tap Water Sampling and Environmental Implications

To evaluate the reduction performance of environmental substances, humic acid was introduced into the systems at concentrations ranging from 1 g L^−1^ to 7 g L^−1^. The initial concentration of NDMA was 500 μg L^−1^, the dosage of FAC-600 was maintained at 0.5 g L^−1^, and the dosage of PS was 0.8 mmol L^−1^, which was consistent with the batch experiment setup. Tap water samples were collected from four major cities in China. All samples were collected using 500 mL polypropylene bottles and were subsequently filtered through a 0.45 μm hydrophilic polyethersulfone membrane. The filtered supernatant was stored at 4 °C for preservation. The tap water samples were then spiked with NDMA to achieve an initial concentration of 500 μg L^−1^. The quantitative analysis of NDMA was performed using the UPLC, following the method described in Section 2.3 of this study.

### 2.6. Characterization

The morphology of the nanomaterials was observed using a German ZEISS Sigma (Oberkochen, Germany) 300 scanning electron microscope (SEM). The fine structure was observed by an FEI Talos F200x transmission electron microscope (TEM). The crystal structure of the nanomaterial was observed by Bruker D8 ADVANCE X-ray diffraction (XRD; Bruker, Billerica, MA, USA). Fourier-transform infrared (FTIR) spectra were measured using a Thermo Fisher Scientific (Waltham, MA, USA) Nicolet iS20 in the range of 400–4000 cm^−1^ to determine chemical groups. The Brunauer–Emmett–Teller (BET) and Barrett–Joyner–Halenda (BJH) equations were used with a Quantachrome SI-MP to calculate the surface area and pore size distribution. X-ray photoelectron spectroscopy (XPS) spectra were recorded in a Thermo Scientific K-Alpha to determine surface elemental composition and valency. The zeta potential was measured using a zeta potentiometer (British Malvern Zetasizer, Worcestershire, UK).

## 3. Results

### 3.1. Creation of One-Step-Carbonized FAC

**Figure 1:**
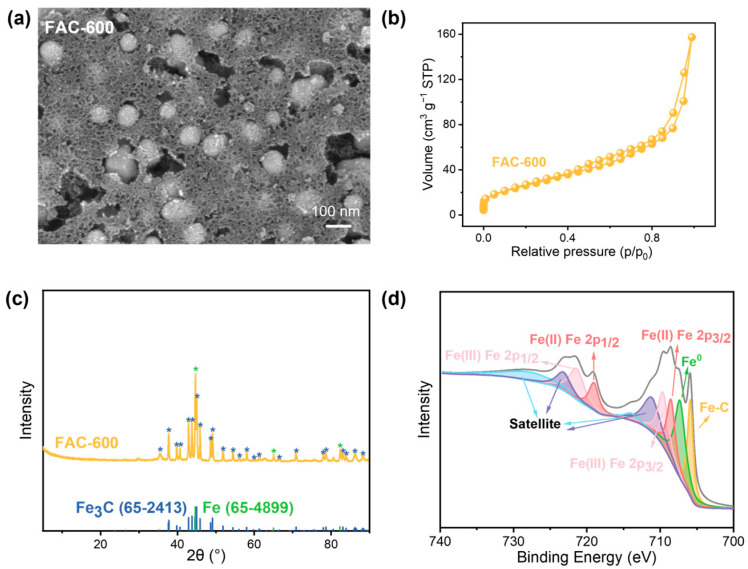
The SEM images (**a**), nitrogen adsorption–desorption curve (**b**), XRD spectra (**c**), and Fe 2p core region (**d**) of FAC-600.

In order to establish an effective approach for the degradation of NDMA, ferric ammonium citrate (FAC) was carbonized through a one-step pyrolysis process at 600 °C, resulting in the formation of an FAC-600 catalyst for the AOP. SEM images (Figure 1a) illustrate that the carbonized FAC exhibits a combination of nanosheet and spherical morphologies, with nanospheres having a diameter of approximately 200 nm. This unique morphology contributes to the high specific surface area of FAC-600. The BET surface area of FAC-600 was determined to be 98.2 m^2^ g^−1^, with a pore volume of 0.24 cm^3^ g^−1^. The nitrogen adsorption isotherm curve indicates that FAC-600 follows the shape of a type IV isotherm with a hysteresis loop, displaying a horizontal plateau post the filling of mesopores (Figure 1b). As demonstrated in Figure S3, the pore size of FAC-600 is concentrated in the range of mesoporous. Although nonstructured mesopores may limit mass transfer efficiency, a moderate pore size (>2 nm) still ensures the diffusion of NDMA molecules (NDMA molecule diameter of 0.45 nm) [1].

Energy-dispersive X-ray spectroscopy (EDS) elemental mapping results (Figure S4) reveal minimal differences in elemental composition between the various morphologies. XRD spectra (Figure 1c) suggest that the one-step pyrolysis method employed in this study results in the formation of ZVI and Fe_3_C within FAC-600, in line with the high iron content observed in the EDS analysis. The presence of ZVI and Fe_3_C in FAC-600 was further confirmed by XPS analysis (Figure 1d). The Fe 2p peak fitting results in the XPS spectrum show peaks corresponding to ZVI and Fe-C functional groups at 705.9 eV and 707.3 eV, respectively. These findings demonstrate the successful fabrication of ZVI and Fe_3_C within the FAC-600 nanomaterials using the proposed pyrolysis method. In addition, Fe(II) and Fe(III) were also observed.

### 3.2. NDMA Removal Behaviors

#### 3.2.1. Catalytic Effect and Reaction Kinetics of FAC-600 in NDMA Degradation

**Figure 2:**
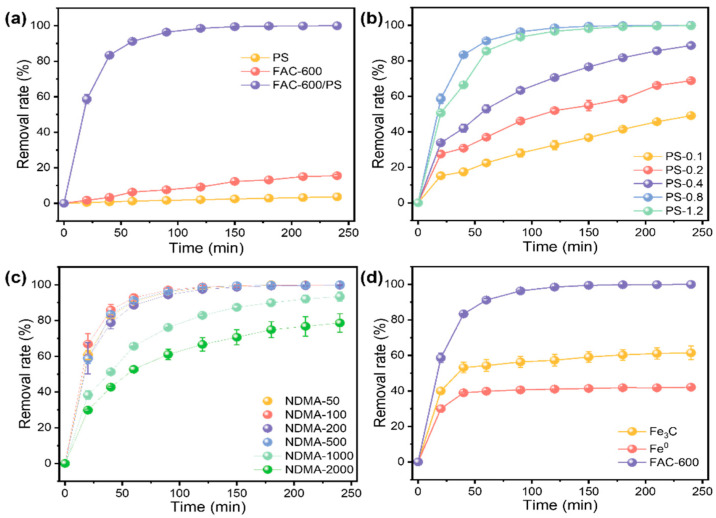
(**a**) The removal rate of NDMA with the PS, FAC-600, and FAC-600/PS systems. (**b**) The removal rate of NDMA with the FAC-600/PS system with different PS concentrations. (**c**) The removal rate of NDMA with the FAC-600/PS system with different NDMA concentrations. (**d**) The removal rate of NDMA with the Fe_3_C/PS, Fe^0^/PS, and FAC-600/PS systems. The initial concentration of NDMA is 500 μg L^−1^. The dosage of FAC-600 is 0.5 g L^−1^. The error bars represent the standard deviation of data from three distinct samples (*n*  =  3).

In order to assess the catalytic performance of FAC-600 in the AOP for NDMA degradation, the removal behavior of NDMA was investigated under various systems (Figure 2a). Initially, in the system containing PS and NDMA, it was observed that only a limited amount of NDMA was removed. This suggests that PS alone struggles to effectively oxidize NDMA. Upon the introduction of FAC-600 into the NDMA system without PS, a limited 15.5% removal rate was achieved within 240 min. In the comparison results of NDMA removal in atmospheric and anaerobic conditions (Figure S5), FAC-600 possesses limited capability to directly reduce or adsorb NDMA under the atmospheric condition in this study. Notably, when FAC-600 and PS were introduced together, the removal rate of NDMA exceeded 90% within 60 min, demonstrating that FAC-600 can enhance the advanced oxidation system of PS for NDMA removal. The reaction kinetics of these three systems further confirmed the high NDMA removal efficiency of FAC-600/PS, with a kinetic rate constant (k_obs_) reaching 0.034 min^−1^, compared to 0.00072 min^−1^ and 0.00015 min^−1^ for the PS and FAC-600 systems, respectively (Figure S6) These results demonstrate the significant catalytic effect of FAC-600 in the PS system with the presence of oxygen.

Moreover, the NDMA removal rate and kinetics at different PS concentrations were investigated, as depicted in Figure 2b and Figure S7. It was observed that as the PS concentration increased from 0.1 mmol L^−1^ to 1.2 mmol L^−1^, k_o__bs_ gradually rose from 0.0026 min^−1^ to 0.025 min^−1^, with the highest kobs of 0.034 min^−1^ noted at a PS concentration of 0.8 mmol L^−1^. Higher concentrations of persulfate can provide ample oxidants to generate free radicals, thereby facilitating the efficient degradation of NDMA. However, excessively high concentrations of sodium persulfate can lead to the rapid generation of a large amount of radicals, which may undergo quenching reactions, such as 2SO_4_•^−^→ S_2_O_8_^²−^ [9], reducing the effective concentration of radicals and, consequently, diminishing interaction and reaction opportunities with the target NDMA. This observation aligns with the decrease in k_obs_ noted at a PS concentration of 1.2 mmol L^−1^.

Furthermore, the removal performance of different concentrations of NDMA with FAC-600 is presented in Figure 2c. Nearly 100% of NDMA could be removed within 120 min when the initial concentration of NDMA was below 500 μg L^−1^, with an FAC-600 dosage of 0.5 g L^−1^ and a PS concentration of 0.8 mmol L^−1^. These results underscore the effective NDMA removal capacity of the proposed PS-based AOP system catalyzed by FAC-600. Additionally, the removal rate gradually decreased with the increase in NDMA’s initial concentration, attributed to competitive effects and the consumption of oxidants [17]. However, even at an initial NDMA concentration of 2000 μg L^−1^, 78.6% of NDMA could still be removed within 240 min.

To elucidate the individual roles of Fe_3_C and ZVI, AOP reactions were carried out using their respective commercial nanomaterials as catalysts (Figure 2d). The findings demonstrate that both Fe_3_C and ZVI are effective in NDMA removal, with Fe_3_C exhibiting relatively superior reaction kinetics and achieving a higher removal rate within a 240 min reaction time. However, it is noteworthy that removal efficiency is limited when using these single nanomaterials. This limitation indicates that the proposed composite material, FAC-600, further enhances the efficiency of the AOP reaction. The NDMA removal capacity of this proposed method is comparable with reported results (Table S2).

#### 3.2.2. Effect of pH and Anions on NDMA Removal

**Figure 3:**
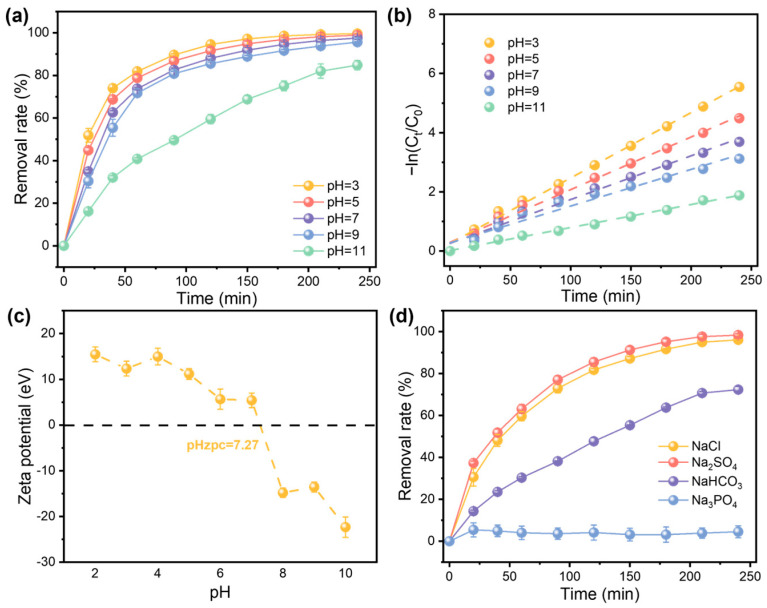
The removal rate (**a**) and reaction kinetics (**b**) of NDMA with the FAC-600/PS system with different pH values. (**c**) The ζ-potential of FAC-600 as a function of pH value. (**d**) The removal rate of NDMA with the FAC-600/PS system with different salts. The initial concentration of NDMA is 500 μg L^−1^. The dosage of FAC-600 is 0.5 g L^−1^. The error bars represent the standard deviation of data from three distinct samples (*n*  =  3).

The pH value of the AOP system significantly affects NDMA removal efficiency. As demonstrated in Figure 3a, the reaction kinetics gradually decrease with the increase in the initial pH value of the NDMA solution. When the initial pH value reaches 11, the k_obs_ of the AOP system reduces to 0.0078 min⁻^1^ (Figure 3b). Zeta potential measurements (Figure 3c) reveal that the point of zero charge (pH_zpc_) of FAC-600 is 7.27, suggesting that the surface charge of FAC-600 is negative when the system pH is higher than 7.27. Notably, k_obs_ was only slightly reduced from pH 3 to pH 9, which is likely related to the regulating effect on pH value of sodium persulfate [18]. Diethylamine, a typical organic base functional group with a pKa of 10.7, indicates that NDMA tends to dissociate to a negatively charged species at pH levels higher than 10.7. This dissociation results in charge repulsion between the negatively charged NDMA and the negatively charged FAC-600 surface [13], thereby reducing the efficiency of the AOP reaction. Moreover, a lower-pH environment (pH < 11) is conducive to the protonation of NDMA and the stability of PS [19], which promotes the occurrence of AOP reactions by facilitating the interaction between NDMA and the oxidizing species generated in the system.

Inorganic anions such as chloride, carbonate, phosphate, and sulfate are ubiquitous in water. They react with radicals produced during AOPs to form chlorine radicals, carbonate radicals, phosphate radicals, and sulfate radicals, respectively, which significantly influence the transformation of organic pollutants [19]. These effects were also demonstrated in this study, as illustrated in Figure 3d. Four types of anionic sodium salts were added to the reaction system at a concentration of 5 mmol L^−1^. Compared with the pure water system, the reaction kinetics constant of NDMA decreased significantly, with the ranking of k_obs_ as follows: Na_2_SO_4_ > NaCl > NaHCO_3_ > Na_3_PO_4_ (Figure S8). It is worth noting that the introduction of bicarbonate and phosphate salts increases the solution pH, which affects the stability of PS and consequently inhibits the degradation of NDMA [19]. Additionally, phosphate ions can form complexes with transition metal ions such as Fe(II). As revealed in Section 3.1, Fe(II) was characterized in FAC-600, indicating the presence of Fe(II)-induced AOPs in the NDMA removal system. The cycle between Fe(II) and Fe(III) was proven to be key for catalytic stability. Therefore, the complexation reaction affects the Fe(II)/Fe(III) cycle, further impacting the performance of AOPs and resulting in a limited removal capacity of NDMA with the presence of Na_3_PO_4_.

### 3.3. Mechanisms for Removal of NDMA

**Figure 4:**
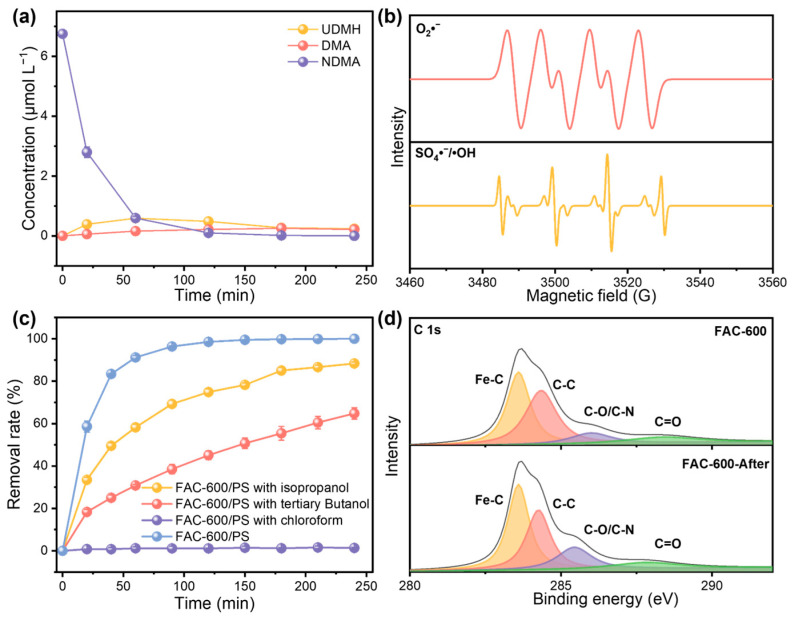
(**a**) The concentration of NDMA, DMA, and UDMH during the AOP. (**b**) EPR spectra of free radicals in FAC-600/PS system for NDMA removal. (**c**) The removal rate of NDMA with the presence of salts. The initial concentration of NDMA is 500 μg L^−1^. The dosage of FAC-600 is 0.5 g L^−1^. The error bars represent the standard deviation of data from three distinct samples (*n*  =  3). (**d**) XPS spectra (C 1s core region) of FAC-600 before and after NDMA removal.

To elucidate the mechanisms underlying the oxidative degradation of NDMA by the FAC-600/AOP system, we investigated the reduction products generated during the NDMA removal process. Due to the strong reduction properties of ZVI and Fe_3_C, we monitored these products throughout the experiment. As illustrated in Figure 4a, limited amounts of DMA and UDMH were detected within the 240 min reaction period, despite significant NDMA removal. This observation suggests that the direct reduction of NDMA by FAC-600 is inhibited in the FAC-600/AOP system, as indicated by the low concentrations of DMA and UDMH and the high k_obs_ for NDMA removal (Figure S6).

To further elucidate the NDMA removal mechanism in the FAC-600/PS AOP system, we employed Electron Paramagnetic Resonance (EPR) characterization. As shown in Figure 4b, superoxide radicals (O_2_•^−^), hydroxyl radicals (•OH), and sulfate radicals (SO_4_•^−^) were observed during the AOP reaction for NDMA degradation [20]. Although the magnetic properties of FAC introduce strong background noise interference in the EPR results, the fitting results still demonstrate that the signal peak of sulfate radicals in the system is relatively low. This observation was further corroborated by radical quenching experiments (Figure 4c). In these experiments, isopropanol, tertiary butanol, and trichloromethane were used as quenchers for sulfate, hydroxyl, and superoxide radicals, respectively [21]. The results indicated that the addition of trichloromethane significantly affected NDMA removal efficiency, whereas the addition of isopropanol only moderately reduced NDMA removal. This suggests that ·O_2_•^−^ plays a significant role in NDMA degradation. The presence of dissolved oxygen in AOP-treated water containing low levels of contaminants likely leads to the formation of superoxide radicals. Initially, any reducing hydrated electrons formed would be consumed by the dissolved oxygen, resulting in the generation of superoxide radicals [22].

The presence of ZVI, Fe_3_C, and Fe(II) in FAC-600 facilitates a redox cycle between Fe(II) and Fe(III), which is crucial for generating these reactive radicals. Additional characterization of FAC-600 before and after the AOP reaction supports this hypothesis. XRD spectra (Figure S9) show a decrease in the ZVI content of FAC-600 after the reaction, consistent with the reduction mechanism that forms Fe(II) in AOP reactions [23]. Notably, this decrease remains limited even after the 240 min reaction time, indicating the synergy between Fe_3_C and ZVI. ZVI, a potent reducing agent, directly facilitates electron activation for PS but is prone to oxidation and deactivation [5]. The distinctive electronic structure of Fe_3_C involving metal–carbon bonds could stabilize Fe species, enhancing electron cycling and preventing surface passivation of ZVI (Figure S10). Both XPS and FTIR analyses further confirm the presence of C-O/C-N functional groups in FAC-600 (Figure 4d and Figure S11), which help prevent the rapid depletion of ZVI and facilitate electron transfer [15]. The relative content of C-O/C-N functional groups in FAC-600 significantly changed after the reaction. As reported in a previous study, this indicates that carbon-based functional groups may play a role during electron transfer processes [24].

As demonstrated by the characterization results, the presence of oxygen in the reaction system may lead to the generation of superoxide radicals through the reduction of ZVI. Due to the reducibility of O_2_•^−^ and the low electron cloud density of NDMA molecules, this system is conducive to the direct degradation of NDMA [25]. Based on the limited concentration of DMA and UDMA in this AOP system, the rapid formation of hydroxyl and sulfate radicals in the ZVI/PS system may promote the degradation of NDMA and its reduction by-products, resulting in a high k_obs_. Compared to commercial ZVI and Fe_3_C, the carbon-based functional groups in FAC-600 may effectively enhance electron transfer and mitigate the rapid consumption of ZVI, ensuring sustained catalytic activity throughout the reaction.

### 3.4. Environmental Implications

**Figure 5:**
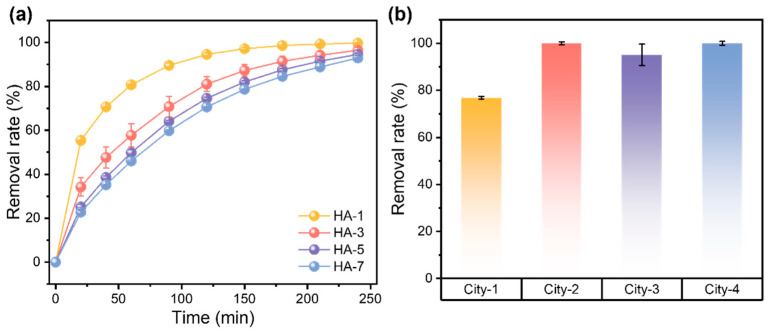
(**a**) The removal rate of NDMA with the FAC-600/PS system with different concentrations of HA. (**b**) The removal rate of NDMA with practical tap water matrices. The initial concentration of NDMA is 500 μg L^−1^. The dosage of FAC-600 is 0.5 g L^−1^. The error bars represent the standard deviation of data from three distinct samples (*n*  =  3).

As demonstrated by mechanism elucidation, FAC-600 exhibited efficient NDMA removal capacity across a wide range of concentrations. The stability and reusability of FAC-600 were assessed over three cycles. After each 240 min reaction, FAC-600 was collected, rinsed with ultrapure water, and dried in preparation for the next cycle. As demonstrated in Figure S12, the effectiveness of FAC-600/PS in removing NDMA slightly decreases over three testing cycles, indicating considerable stability as a catalyst. Moreover, with its one-step fabrication method, FAC-600 provides a novel and convenient approach for degrading NDMA in contaminated water. Given that NDMA is a significant contaminant in drinking water, often forming as a by-product of disinfection processes such as chlorination or ozonation, it is essential to evaluate the reduction performance of NDMA within practical matrices. To assess these effects, HA was introduced into the systems at concentrations ranging from 1 g L^−1^ to 7 g L^−1^, as HA is commonly found in drinking water and is known to interfere with the disinfection process. As illustrated in Figure 5a, more than 90% of the NDMA was removed within 240 min, even in the presence of 7 g/L HA. However, the reaction kinetics slightly decreased with increasing HA concentration, likely due to the competitive effect of reducing functional groups in HA on free radicals.

To further evaluate the removal performance of the proposed FAC-600/PS AOP system in real-world drinking water matrices, tap water samples were collected from four different cities across China (Table S1). For a consistent comparison of degradation performance across various matrices, the NDMA concentration was adjusted to 500 μg L^−1^ in each sample. The reduction results (Figure 5b) show that NDMA was effectively removed, achieving an average removal rate of 93.2% in the four types of drinking water matrices within 240 min. The high removal rate of NDMA in all four samples indicates that FAC-600 maintained significant catalytic efficiency even under the complex conditions posed by disinfection processes. It is noteworthy that a slight reduction in both the reaction kinetics and capacity was observed in practical drinking water matrices compared to the ultrapure water matrix, suggesting a competitive reaction phenomenon between other reductants present in drinking water.

## 4. Discussion

In this study, we aimed to propose a feasible method for the efficient removal of NDMA from contaminated drinking water. Compared to reported composite catalysts, FAC-600, fabricated through the one-step carbonization of ferric ammonium citrate, significantly reduces the complexity of manufacturing processes and associated high costs. The catalytic effects of ZVI and Fe_3_C generated during the one-step carbonization process, along with the electron transfer ability of the C/N structure, enable FAC-600 to efficiently catalyze the AOP system, achieving the rapid degradation of NDMA through superoxide and hydroxyl radicals. Using a limited dosage of FAC-600 (0.5 g L^−1^), the proposed FAC-600/PS AOP system achieved more than 90% NDMA removal across a wide concentration range (50 μg L^−1^ to 1000 μg L^−1^). Additionally, the buffering capacity of PS allows this AOP system to maintain good stability in NDMA degradation, with effective performance across an initial pH range of 3 to 9.

Mechanistic insights reveal that O_2_•^−^ plays a dominant role in the degradation of NDMA within the FAC-600/PS AOP system. The presence of dissolved oxygen tends to consume reducing hydrated electrons, thereby generating superoxide radicals. Compared to other free radicals, superoxide radicals exhibit higher reducibility and a longer half-life, making them more effective in reacting with NDMA, which is further degraded by the abundant hydroxyl radicals present in the system. This mechanism enables the system to efficiently and stably remove NDMA in complex drinking water matrices.

Overall, these findings highlight the critical role of catalysts like FAC-600, which are produced through a one-step fabrication method, in advancing AOP technologies for environmental remediation. The FAC-600/AOP system serves as a robust and efficient approach for NDMA removal, exhibiting high catalytic efficiency across various water matrices, including those containing interfering substances like humic acid. The system’s impressive performance in real-world drinking water conditions underscores its potential applicability for mitigating NDMA contamination, thereby contributing to safer drinking water.

## Data Availability

The data supporting the findings of the study are included in the main text and Supplementary Materials. Additional data are available from the corresponding author upon request.

## Supplementary Materials

The following supporting information can be downloaded at https://www.mdpi.com/article/10.3390/nano15110831/s1, Figure S1: Chemical structures of DMA and UDMH; Figure S2: The removal rate of NDMA with PS-based AOPs system using FAC as catalyst under the carbonization temperature of 600 °C, 800 °C, and 1000 °C; Figure S3: Pore size distribution results of FAC-600; Figure S4: Energy-dispersive X-ray spectroscopy (EDS) elemental mapping results of FAC-600; Figure S5: Comparison results of NDMA removal with FAC-600 under atmospheric and anaerobic conditions; Figure S6: The reaction kinetics of the three systems for NDMA removal: PS, FAC-600, and FAC-600/PS; Figure S7: The reaction kinetics of FAC-600/PS systems for NDMA removal with different PS concentrations; Figure S8: The reaction kinetics of FAC-600/PS systems for NDMA removal with different anions; Figure S9: XRD spectra of FAC-600 and FAC-600 after AOP reaction; Figure S10: Schematic of the synergy mechanism between Fe3C and ZVI; Figure S11: FTIR spectra of FAC-600 and FAC-600 after AOP reaction; Figure S12: The reusability of FAC-600 over three cycles; Table S1: Characteristics of 4 typical tap water samples in this study; Table S2: Performance comparison of NDMA removal. References [26,27,28] are cited in the supplementary materials.

## Abbreviations

The following abbreviations are used in this manuscript:

NDMA	N-Nitrosodimethylamine
AOPs	Advanced oxidation processes
FAC	Ferric ammonium citrate
PS	Persulfate
NF	Nanofiltration
RO	Reverse osmosis
DMA	Dimethylamine
UDMH	Unsymmetrical dimethylhydrazine
UV	Ultraviolet
ZVI	Zero-valent iron
HA	Humic acid
UPLC	Liquid chromatograph
GC	Gas chromatography
MS	Mass spectrometry
SIM	Selected ion mode
SEM	Scanning electron microscope
TEM	Transmission electron microscope
XRD	X-ray diffraction
FTIR	Fourier-transform infrared
BET	Brunauer–Emmett–Teller
BJH	Barrett–Joyner–Halenda
XPS	X-ray photoelectron spectroscopy
EDS	Energy-dispersive X-ray spectroscopy
EPR	Electron paramagnetic resonance

## References

[1] Sgroi M., Vagliasindi F.G.A., Snyder S.A., Roccaro P. (2018). N-Nitrosodimethylamine (NDMA) and its precursors in water and wastewater: A review on formation and removal. Chemosphere.

[2] Ersan M.S., Ladner D.A., Karanfil T. (2015). N-Nitrosodimethylamine (NDMA) Precursors Leach from Nanofiltration Membranes. Environ. Sci. Technol. Lett..

[3] Lee C., Yoon J., Von Gunten U. (2007). Oxidative degradation of N-nitrosodimethylamine by conventional ozonation and the advanced oxidation process ozone/hydrogen peroxide. Water Res..

[4] Szczuka A., Huang N., MacDonald J.A., Nayak A., Zhang Z., Mitch W.A. (2020). N-Nitrosodimethylamine Formation during UV/Hydrogen Peroxide and UV/Chlorine Advanced Oxidation Process Treatment Following Reverse Osmosis for Potable Reuse. Environ. Sci. Technol..

[5] Qin H., Guan X., Tratnyek P.G. (2019). Effects of Sulfidation and Nitrate on the Reduction of N-Nitrosodimethylamine by Zerovalent Iron. Environ. Sci. Technol..

[6] Han Y., Wang J., Li J., Chen Z., Li W., Jiang B., Yao J. (2018). Copper Corrosion Products Catalyzed Reduction of N-Nitrosodimethylamine with Iron. Environ. Sci. Technol..

[7] Fujioka T., Masaki S., Kodamatani H., Ikehata K. (2017). Degradation of N-Nitrosodimethylamine by UV-Based Advanced Oxidation Processes for Potable Reuse: A Short Review. Curr. Pollut. Rep..

[8] Roback S.L., Ishida K.P., Chuang Y.-H., Zhang Z., Mitch W.A., Plumlee M.H. (2021). Pilot UV-AOP Comparison of UV/Hydrogen Peroxide, UV/Free Chlorine, and UV/Monochloramine for the Removal of N-Nitrosodimethylamine (NDMA) and NDMA Precursors. ACS EST Water.

[9] Velo-Gala I., Farré M.J., Radjenovic J., Gernjak W. (2019). N-Nitrosodimethylamine (NDMA) Degradation by the Ultraviolet/Peroxodisulfate Process. Environ. Sci. Technol. Lett..

[10] Yan Y., Wei Z., Duan X., Long M., Spinney R., Dionysiou D.D., Xiao R., Alvarez P.J.J. (2023). Merits and Limitations of Radical vs. Nonradical Pathways in Persulfate-Based Advanced Oxidation Processes. Environ. Sci. Technol..

[11] Liu N., Zhang L., Xue Y., Lv J., Yu Q., Yuan X. (2017). Nitrogen-doped carbon material as a catalyst for the degradation of direct red23 based on persulfate oxidation. Sep. Purif. Technol..

[12] Zhang S., Hedtke T., Zhu Q., Sun M., Weon S., Zhao Y., Stavitski E., Elimelech M., Kim J.-H. (2021). Membrane-Confined Iron Oxychloride Nanocatalysts for Highly Efficient Heterogeneous Fenton Water Treatment. Environ. Sci. Technol..

[13] Zhang L., Li M., Tang C., Wang H., Zhang X., Wang J., Li H., Mahtab M.S., Yue D. (2024). Mechanistic Insights into the Removal of Surfactant-Like Contaminants on Mesoporous Polydopamine Nanospheres from Complex Wastewater Matrices. Environ. Sci. Technol..

[14] Kong X., Wang J., Zheng K., Shao Y., Cui D., Wang C., Zhang L., Jiang B., Wang C., Yue D. (2025). Deciphering the transport, retention, and mechanisms of stabilized sulfidated microscale zerovalent iron for in situ remediation of vanadium (V). Sep. Purif. Technol..

[15] Feng H., Tang L., Tang J., Zeng G., Dong H., Deng Y., Wang L., Liu Y., Ren X., Zhou Y. (2018). Cu-Doped Fe@Fe_2_O_3_ core–shell nanoparticle shifted oxygen reduction pathway for high-efficiency arsenic removal in smelting wastewater. Environ. Sci. Nano.

[16] Li J., Qin H., Guan X. (2015). Premagnetization for Enhancing the Reactivity of Multiple Zerovalent Iron Samples toward Various Contaminants. Environ. Sci. Technol..

[17] Zhou H., Zhong S., Chen J., Ren S., Ren W., Lai B., Guan X., Ma T., Wang S., Duan X. (2024). Overlooked Complexation and Competition Effects of Phenolic Contaminants in a Mn(II)/Nitrilotriacetic Acid/Peroxymonosulfate System: Inhibited Generation of Primary and Secondary High-Valent Manganese Species. Environ. Sci. Technol..

[18] Zhang X., Zhang Y., Tian J., Guo Y., Zhou Z., Liu Z., Zhao Z., Liu B., Li J. (2024). Generating ^1^O_2_ and CoIV=O through efficient peroxymonosulfate activation by ZnCo_2_O_4_ nanosheets for pollutant control. Nano Res..

[19] Wang J., Wang S. (2021). Effect of inorganic anions on the performance of advanced oxidation processes for degradation of organic contaminants. Chem. Eng. J..

[20] Hicks R.G., Hooper R. (1999). Synthesis and EPR Characterization of “Phosphaverdazyl” Radicals. Inorg. Chem..

[21] Wang J., Wang S. (2020). Reactive species in advanced oxidation processes: Formation, identification and reaction mechanism. Chem. Eng. J..

[22] Zhang C., Li T., Zhang J., Yan S., Qin C. (2019). Degradation of p-nitrophenol using a ferrous-tripolyphosphate complex in the presence of oxygen: The key role of superoxide radicals. Appl. Catal. B Environ..

[23] Ferreira M.B., Muñoz-Morales M., Sáez C., Cañizares P., Martínez-Huitle C.A., Rodrigo M.A. (2020). Improving biotreatability of hazardous effluents combining ZVI, electrolysis and photolysis. Sci. Total Environ..

[24] Ding L., Zhang P., Luo H., Hu Y., Norouzi Banis M., Yuan X., Liu N. (2018). Nitrogen-Doped Carbon Materials as Metal-Free Catalyst for the Dechlorination of Trichloroethylene by Sulfide. Environ. Sci. Technol..

[25] Luo Z., Yan Y., Spinney R., Dionysiou D.D., Villamena F.A., Xiao R., Vione D. (2024). Environmental implications of superoxide radicals: From natural processes to engineering applications. Water Res..

[26] Dai X., Zou L., Yan Z., Millikan M. (2009). Adsorption characteristics of N-nitrosodimethylamine from aqueous solution on surface-modified activated carbons. J. Hazard. Mater..

[27] Zhu J.H., Yan D., Rong Xai J., Ma L.L., Shen B. (2001). Attempt to adsorb N-nitrosamines in solution by use of zeolites. Chemosphere.

[28] Lin L., Xu B., Lin Y.-L., Yan L., Shen K.-Y., Xia S.-J., Hu C.-Y., Rong R. (2013). Reduction of N-Nitrosodimethylamine (NDMA) in Aqueous Solution by Nanoscale Fe/Al_2_(SO_4_)_3_. Water Air Soil Pollut..

