# Supplementary material for: Enhancing Catalytic Removal of N-Nitrosodimethylamine from Drinking Water Matrices with One-Step-Carbonized Ferric Ammonium Citrate
Source: Nanomaterials (Basel). 2025 May 30;15(11):831. doi: 10.3390/nano15110831 (PMC12157025; doi:10.3390/nano15110831)
Supplement: Supplementary file 1 [file nanomaterials-15-00831-s001.zip › nanomaterials-3657695-supplementary.pdf]

SUPPORTING INFORMATION

Enhancing catalytic removal of N-Nitrosodimethylamine with one-step carbonized ferric ammonium citrate from drinking water matrices

Jing Lv[1], Lingyue Zhang[2,*], Jialu Li[1], Yuting Zhang[4], Ruofan Wang[1], Rui Tang[1], Jianchao Wang[5], Mei Hong[1], Na Liu[3,*]

1 Key Laboratory of Groundwater Resources and Environment, Ministry of Education, College of New Energy and Environment, Jilin University, Changchun 130021, China
2 Department of Civil Engineering, The University of Hong Kong, Pokfulam, Hong Kong SAR, China
3 Department of Ecology, College of Life Science and Technology, Jinan University, Guangzhou, 510632, Guangdong, China
4 Key Laboratory of Songliao Aquatic Environment, Ministry of Education, Jilin Jianzhu University, Changchun 130118, China
5 State Key Laboratory of Nutrient Use and Management, College of Resources and Environmental Sciences, National Academy of Agriculture Green Development, Key Laboratory of Plant–Soil Interactions (Ministry of Education), China Agricultural University, Beijing 100193, China.

The supporting information consists of 18 pages, 12 figures and 2 table.

Content

Figure S1. Chemical structures of DMA and UDMH.

Figure S2. The removal rate of NDMA with PS-based AOPs system using FAC as catalyst under the carbonization temperature of 600 ℃, 800 ℃, and 1000 ℃

Figure S3. Pore size distribution results of FAC-600.

Figure S4. Energy dispersive X-ray spectroscopy (EDS) elemental mapping results of FAC-600.

Figure S5. Comparison results of NDMA removal with FAC-600 under atmospheric and anaerobic conditions.

Figure S6. The reaction kinetics of these three systems for NDMA removal: PS, FAC-600, and FAC-600/PS.

Figure S7. The reaction kinetics of FAC-600/PS systems for NDMA removal with different PS concentration.

Figure S8. The reaction kinetics of FAC-600/PS systems for NDMA removal with different anions.

Figure S9. XRD spectra of FAC-600 and FAC-600 after AOP reaction.

Figure S10. Schematic of the synergy mechanism between Fe3C and ZVI.

Figure S11. FTIR spectra of FAC-600 and FAC-600 after AOP reaction.

Figure S12. The reusability of FAC-600 over three cycles.

Table S1. Characteristics of 4 typical tap water sampled in this study.

Table S2. Performance comparison of NDMA removal.

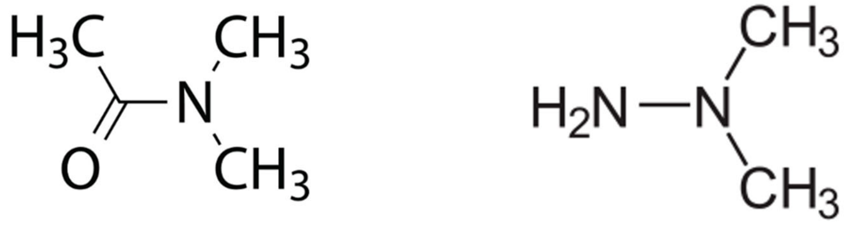

Dimethylamine (DMA) unsymmetrical dimethylhydrazine (UDMH)

Figure S1. Chemical structures of DMA and UDMH.

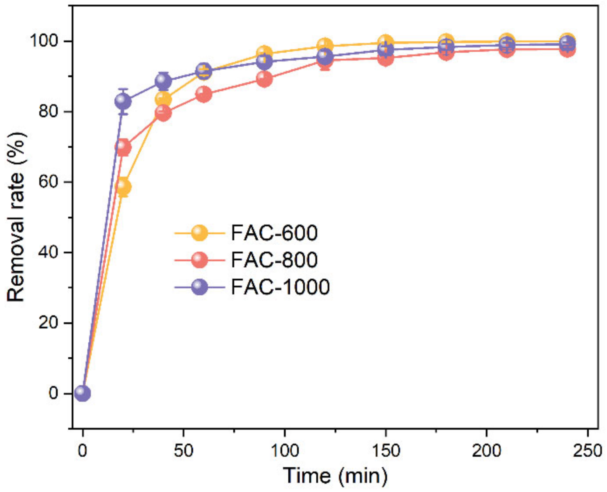

Figure S2. The removal rate of NDMA with PS-based AOPs system using FAC as catalyst under the carbonization temperature of 600 ℃, 800 ℃, and 1000 ℃

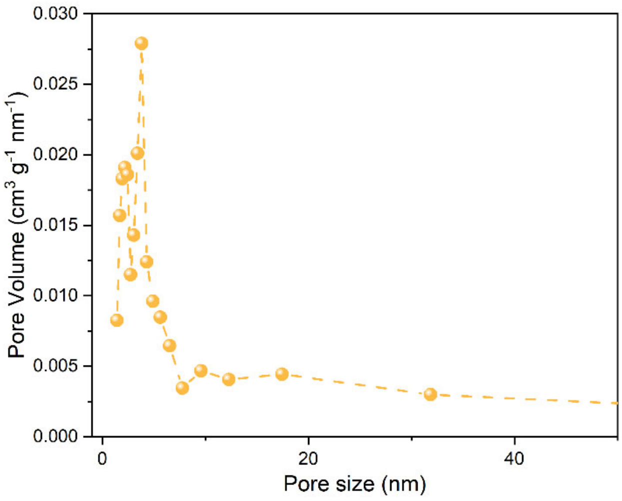

Figure S3. Pore size distribution results of FAC-600.

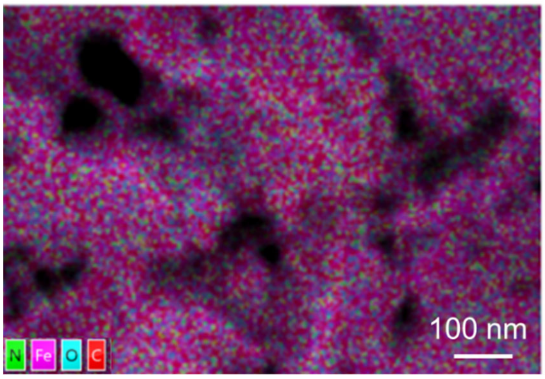

Figure S4. Energy dispersive X-ray spectroscopy (EDS) elemental mapping results of FAC-600.

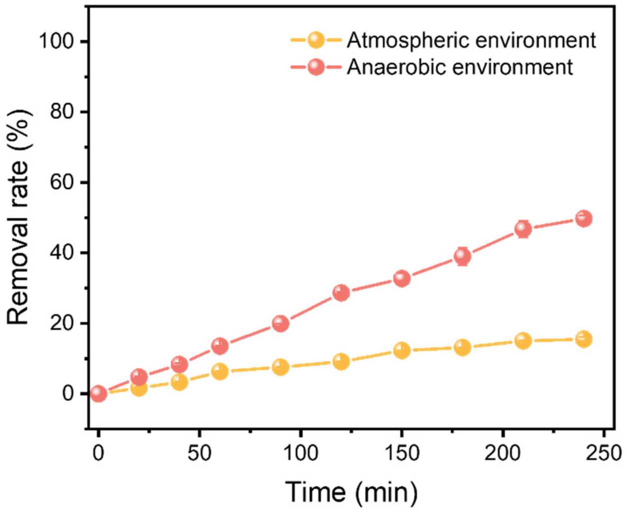

Figure S5. Comparison results of NDMA removal with FAC-600 under atmospheric and anaerobic conditions.

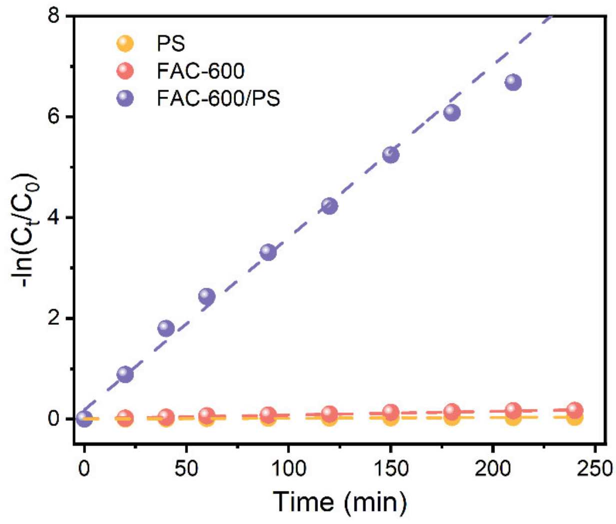

Figure S6. The reaction kinetics of these three systems for NDMA removal: PS, FAC-600, and FAC-600/PS.

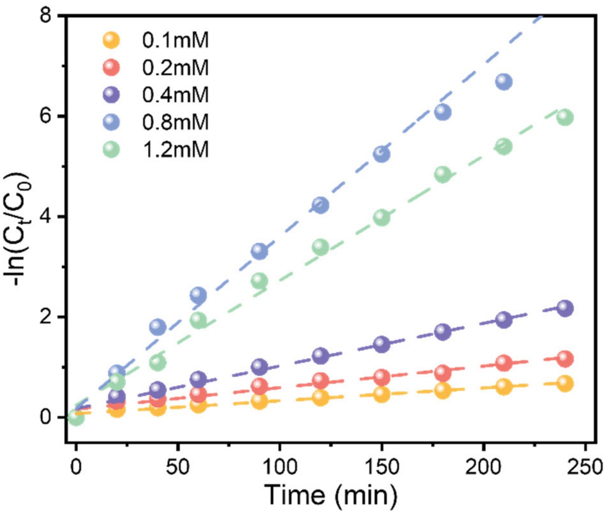

Figure S7. The reaction kinetics of FAC-600/PS systems for NDMA removal with different PS concentration.

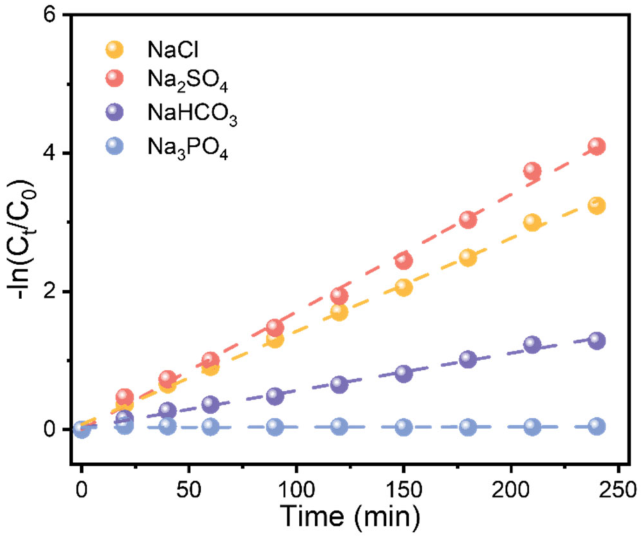

Figure S8. The reaction kinetics of FAC-600/PS systems for NDMA removal with different anions.

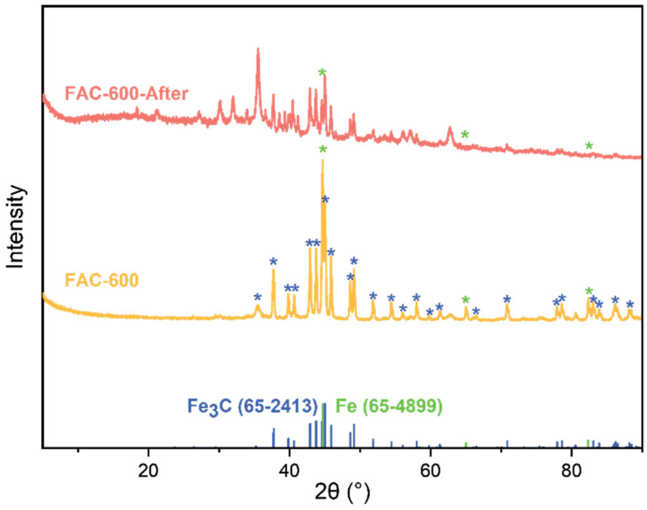

Figure S9. XRD spectra of FAC-600 and FAC-600 after AOP reaction.

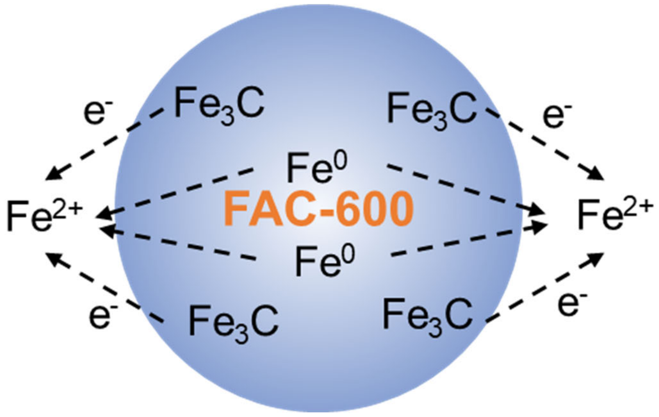

Figure S10. Schematic of the synergy mechanism between Fe_3C and ZVI.

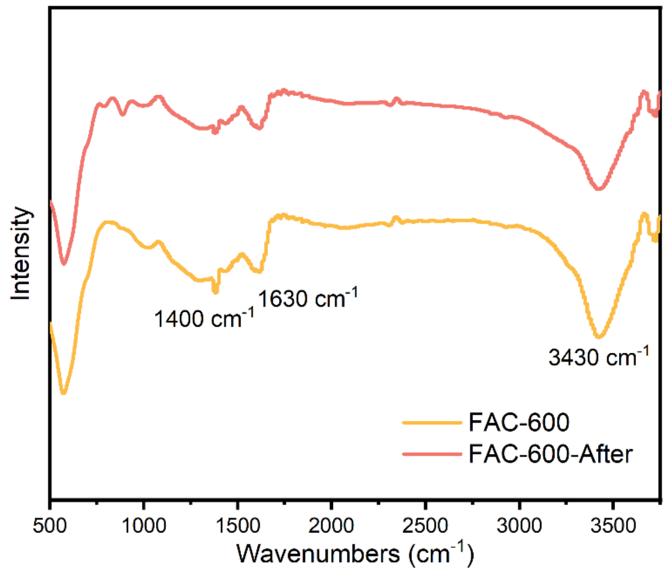

Figure S11. FTIR spectra of FAC-600 and FAC-600 after AOP reaction.

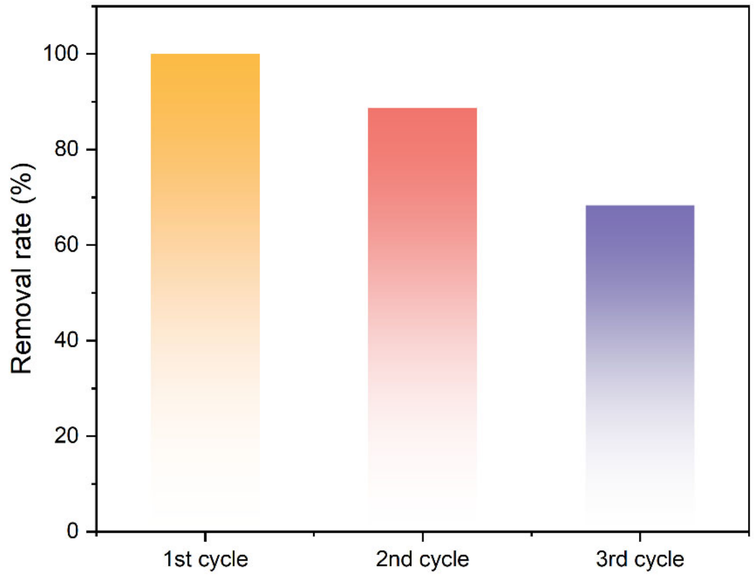

Figure S12. The reusability of FAC-600 over three cycles.

Table S1. Characteristics of 4 typical tap water sampled in this study.

City	pH value	ORP (mV)	Cl^- (mg L^{-1})	COD* (mg L^{-1})
City-1	7.2	170.0	0.17	146.9
City-2	7.5	170.7	0.06	187.3
City-3	6.9	183.8	0.02	114.3
City-4	6.7	325.6	0.07	169.3

* Chemical oxygen demand (COD)

Table S2. Performance comparison of NDMA removal.

Treatment process	Dosage ($g\ L^{-1}$)	Removal rate (%)	Time (min)	Reference
AC-3	1.67	26.7	1440	[1]
Zeolites	20	4.0	1440	[2]
Fe/CuO	10	>99.0	720	[3]
NZVI+$AI_2(SO_4)_3$	5	87.3	1260	[4]
UV Irradiation	2.7 mg/L as Cl_2	81 to 95	240	[5]
FAC-600/PS	0.5	>99.5	120	This work

References:

1. Dai, X.; Zou, L.; Yan, Z.; Millikan, M. Adsorption characteristics of N-nitrosodimethylamine from aqueous solution on surface-modified activated carbons. *Journal of Hazardous Materials* **2009**, *168*, 51-56, doi:https://doi.org/10.1016/j.jhazmat.2009.01.119.
2. Zhu, J.H.; Yan, D.; Rong Xai, J.; Ma, L.L.; Shen, B. Attempt to adsorb N-nitrosamines in solution by use of zeolites. *Chemosphere* **2001**, *44*, 949-956, doi:https://doi.org/10.1016/S0045-6535(00)00560-9.
3. Han, Y.; Wang, J.; Li, J.; Chen, Z.; Li, W.; Jiang, B.; Yao, J. Copper Corrosion Products Catalyzed Reduction of N-Nitrosodimethylamine with Iron. *Environmental Science & Technology* **2018**, *52*, 11735-11742, doi:10.1021/acs.est.8b02574.
4. Lin, L.; Xu, B.; Lin, Y.-L.; Yan, L.; Shen, K.-Y.; Xia, S.-J.; Hu, C.-Y.; Rong, R. Reduction of N-Nitrosodimethylamine (NDMA) in Aqueous Solution by Nanoscale Fe/Al2(SO4)3. *Water, Air, & Soil Pollution* **2013**, *224*, 1632, doi:10.1007/s11270-013-1632-z.
5. Roback, S.L.; Ishida, K.P.; Chuang, Y.-H.; Zhang, Z.; Mitch, W.A.; Plumlee, M.H. Pilot UV-AOP Comparison of UV/Hydrogen Peroxide, UV/Free Chlorine, and UV/Monochloramine for the Removal of N-Nitrosodimethylamine (NDMA) and NDMA Precursors. *ACS ES&T Water* **2021**, *1*, 396-406, doi:10.1021/acsestwater.0c00155.
